# Combining tissue and circulating tumor DNA increases the detection rate of a *CTNNB1* mutation in hepatocellular carcinoma

**DOI:** 10.1186/s12885-021-08103-0

**Published:** 2021-04-08

**Authors:** Stine Karlsen Oversoe, Michelle Simone Clement, Britta Weber, Henning Grønbæk, Stephen Jacques Hamilton-Dutoit, Boe Sandahl Sorensen, Jens Kelsen

**Affiliations:** 1 Department of Hepatology and Gastroenterology, Aarhus University Hospital, Aarhus, Denmark; 2 Department of Internal Medicine, Randers Regional Hospital, Randers, Denmark; 3 Department of Clinical Biochemistry, Aarhus University Hospital, Aarhus, Denmark; 4 Department of Clinical Oncology and Danish Centre of Particle Therapy, Aarhus University Hospital, Aarhus, Denmark; 5 Department of Pathology, Aarhus University Hospital, Aarhus, Denmark

**Keywords:** Hepatocellular carcinoma, Molecular pathology, Circulating tumor DNA, Droplet digital PCR, Predictive biomarkers

## Abstract

**Background and aims:**

Studies suggest that mutations in the *CTNNB1* gene are predictive of response to immunotherapy, an emerging therapy for advanced hepatocellular carcinoma (HCC). Analysis of circulating tumor DNA (ctDNA) offers the possibility of serial non-invasive mutational profiling of tumors. Combining tumor tissue and ctDNA analysis may increase the detection rate of mutations.

This study aimed to evaluate the frequency of the *CTNNB1* p.T41A mutation in ctDNA and tumor samples from HCC patients and to evaluate the concordance rates between plasma and tissue. We further evaluated changes in ctDNA after various HCC treatment modalities and the impact of the *CTNNB1* p.T41A mutation on the clinical course of HCC.

**Methods:**

We used droplet digital PCR to analyze plasma from 95 patients and the corresponding tumor samples from 37 patients during 3 years follow up.

**Results:**

In tumor tissue samples, the mutation rate was 8.1% (3/37). In ctDNA from HCC patients, the *CTNNB1* mutation rate was 9.5% (9/95) in the pre-treatment samples. Adding results from plasma analysis to the subgroup of patients with available tissue samples, the mutation detection rate increased to 13.5% (5/37). There was no difference in overall survival according to *CTNNB1* mutational status. Serial testing of ctDNA suggested a possible clonal evolution of HCC or arising multicentric tumors with separate genetic profiles in individual patients.

**Conclusion:**

Combining analysis of ctDNA and tumor tissue increased the detection rate of *CTNNB1* mutation in HCC patients. A liquid biopsy approach may be useful in a tailored therapy of HCC.

**Supplementary Information:**

The online version contains supplementary material available at 10.1186/s12885-021-08103-0.

## Background

Hepatocellular carcinoma (HCC) is a severe disease with significant morbidity and high mortality [1]. Treatment of HCC is guided by tumor stage, underlying liver disease and performance status. For the past decade, systemic treatment options were limited to sorafenib but recently, other tyrosine-kinase inhibitors demonstrated a survival benefit in advanced HCC [2, 3]. In addition, new treatment strategies with immunotherapy for advanced HCC are emerging [4]. As many treatments will be available for patients with advanced HCC, clinicians need methods to select the right treatment for the right patient.

Pronounced intratumor heterogeneity and the presence of multicentric tumors challenge a global tumor molecular profiling from a single biopsy [5, 6]. In addition, mutational profiling of tumor biopsies taken at an early stage of HCC do not necessarily provide optimal guidance for treatment decisions later in the disease course. Conversely, ctDNA potentially represents the whole tumor burden and may provide information on specific tumor mutations during a clonal evolution. ctDNA analysis is non-invasive and can be performed repeatedly during the course of disease and thereby provide an accurate picture of the tumor mutation profile before clinical decision-making.

*CTNNB1* mutations belong to the most prevalent genetic alterations in HCC [7]. The p.T41A mutation is detected in 10–15% of tissue samples from primary tumors and leads to activation of the Wnt/β-catenin signaling pathway [8]. *CTNNB1* mutations can appear at any time in tumor evolution [9], and Vilarinho et al. suggested a progressive clonal evolution in the *CTNNB1* gene during malignant transformation and development of metastasis [10]. Studies indicate that patients with mutations in the Wnt/β-catenin pathway, including *CTNNB1* mutations, have an inferior response to regorafenib [11] and particularly to immunotherapy [12]. A murine HCC model supports these clinical findings showing resistance to anti-PD-1 therapy in tumors with activation of the Wnt/β-catenin pathway [13].

In the present study we aimed to prospectively monitor and evaluate the concordance between the *CTNNB1* p.T41A mutation in ctDNA and tumor tissue in patients with HCC. For this purpose, droplet digital PCR (ddPCR) is a sensitive, feasible and affordable method for detecting specific low frequency mutated alleles in a background of abundant non-mutated alleles [14]. We hypothesized that the combined analysis of ctDNA and tumor tissue DNA may provide a more complete picture of the frequency of *CTNNB1* mutation. Further, we aimed to evaluate serial testing of ctDNA in a clinical setting and the correlation of the *CTNNB1* mutation to clinical outcome.

## Methods

### Ethical approval

The study was approved by the Central Denmark Region Committee on Biomedical Research Ethics (no. 1–10–72-240-16) and conducted in accordance with the Declaration of Helsinki.

### Patients and sample preparation

The study included 95 patients with the following inclusion criteria; a clinical diagnosis of HCC; plans to initiate a new treatment modality; and age above 18 years and no synchronous malignancy (apart from non-melanoma skin cancer). All patients gave written informed consent. Patients were recruited from November 2016 through October 2018 at the Department of Hepatology and Gastroenterology or the Department of Oncology, Aarhus University Hospital.

Clinical data on disease stage, previous and new treatment(s), treatment response, and mortality were obtained from patient charts.

Blood samples were collected from each patient before commencing a new treatment, after 1 month and every 6 months thereafter for up to 3 years. Samples were collected in 9 mL EDTA tubes (Becton Dickinson, Plymouth, United Kingdom) and processed within 4 h. Samples were centrifuged (1800 g at 4 °C for 10 min), and plasma was carefully removed to avoid contamination and stored at − 80 °C. Tumor tissue was available from 37 of the patients. From 8 patients undergoing resection, fresh tumor tissue samples were collected in liquid nitrogen. All 8 samples passed quality control for mutation analysis. Formalin-fixed, paraffin-embedded (FFPE) samples from 32 previously performed diagnostic biopsies were retrieved for the study and 29 biopsies passed quality control for mutation analysis.

### Cell-free DNA (cfDNA) and tumor tissue DNA analysis

Using QIAamp Circulating Nucleic Acid Kit (Qiagen, Hilden, Germany) cfDNA was extracted from 4 to 5 mL of plasma and eluted in 100 μl of elution buffer. DNA extraction from macro dissected tumor tissue samples was performed with the DNA Mini Kit (Qiagen) on a QIAsymphony according to the manufacturer’s manual.

Doplet digital PCR (ddPCR) analyses were performed with the *CTNNB1* p.T41A and wild type (WT) assay from Bio-Rad using the QX200 AutoDG Droplet Digital PCR system (Bio-Rad, Hercules, CA, USA). Samples were run in triplicates. They underwent quality control and were repeated in cases of outliers or discrepancy. Each run included a nontemplate control, cell-free DNA (cfDNA) from a healthy donor and a mutation-positive control. QuantaSoft analysis software version v.1.7.4.0917 (Bio-Rad) was used in all data analyses.The limit of detection (LoD) was determined as previously described by analyzing cfDNA from healthy donors [15]. See Supplementary material for a more detailed description of the methods used.

### Statistical analysis

Statistical analyses were performed in STATA version 16.0 (Stata Corporation) and GraphPad Prism version 8.3.0. All tests were two-sided, and *P* values < 0.05 were considered statistically significant. Survival analyses were performed using the Cox proportional hazards regression. Correlations between the mutational status and patient characteristics were evaluated by the chi-squared test. Concordance between the mutational status in plasma and tumor tissue was calculated by Cohen’s kappa statistics. Data collection was managed using Research Electronic Data Capture (REDCap) hosted at Aarhus University [16, 17].

## Results

### Patient characteristics

We recruited 95 HCC patients for the study, 24 women (25%) and 71 men (75%). The number of patients with underlying cirrhosis were 61 (64%) and the primary etiology of cirrhosis was alcohol (51%). Median follow-up time was 439 days (IQ range 146–724 days) and 69 (71.5%) patients died within the follow-up period. Characteristics of the patients according to *CTNNB1* mutational status in plasma and tissue can be seen in Table 1. Females were more often *CTNNB1* mutation positive (20.1% vs. 5.6%, *P* = 0.028) and there was a trend towards an association with Hepatitis C virus infection etiology in plasma mutation positive patients (*P* = 0.071). More than half of the patients were in BCLC stage C (57.9%), but tumor load estimated by TNM stage was more evenly distributed in the cohort, reflecting that the BCLC classification includes both factors linked to tumor burden and performance status of the patient.

**Table 1:** Patient characteristics

	Plasma			Tumor tissue		
	CTNNB1 mutation positive	CTNNB1 mutation negative	***P*** value	CTNNB1 mutation positive	CTNNB1 mutation negative	***P*** value
All	(*n* = 9)	(*n* = 86)		(*n* = 3)	(*n* = 34)	
Age	68 (64–82)	72 (66–78)	0.992	65 (55–86)	75 (67–81)	0.559
Sex						
Women	5 (55.6%)	19 (22.1%)	0.028	2 (66.7%)	6 (17.6%)	0.112
Men	4 (44.4%)	67 (77.9%)		1 (33.3%)	28 (82.4%)	
Cirrosis						
Yes	6 (66.7%)	54 (62.8%)	0.918	2 (66.7%)	12 (35.3%)	0.538
No	3 (33.3%)	25 (29.1%)		1 (33.3%)	19 (55.9%)	
Unknown	–	7 (8.1%)		–	3 (8.8%)	
Child Pugh stage						
Noncirrhosis	3 (33.3%)	32 (37.2%)		1 (33.3%)	22 (64.7%)	
A	1 (11.1%)	26 (30.2%)	0.160	1 (33.3%)	7 (20.6%)	0.825
B	5 (55.6%)	23 (26.7%)		1 (33.3%)	5 (14.7%)	
C	–	5 (5.8%)		–	–	
Cirrhosis etiology						
Alcohol	2 (22.2%)	29 (33.7%)	0.802	1 (33.3%)	9 (26.5%)	0.469
Hepatitis C	3 (33.3%)	10 (11.6%)	0.071	1 (33.3%)	3 (8.8%)	0.469
Non alcoholic steatohepatitis	–	4 (4.7%)	0.509	–	1 (2.9%)	0.672
Other (AIH/haemochromatosis)	–	7 (8.1%)	0.374	–	1 (2.9%)	0.672
Unknown/no cirrhosis	4 (44.4%)	36 (41.9%)	0.881	1 (33.3%)	20 (58.8%)	0.393
Barcelona Clinic Liver Cancer stage						
0	2 (22.2%)	9 (10.5%)	0.857	–	2 (5.9%)	0.872
A	1 (11.1%)	13 (15.1%)		1 (33.3%)	9 (26.5%)	
B	1 (11.1%)	9 (10.5%)		–	7 (20.6%)	
C	5 (55.6%)	50 (58.1%)		2 (66.7%)	15 (44.1%)	
D	–	5 (5.8%)		–	1 (2.9%)	
TNM stage						
I	2 (22.2%)	29 (33.7%)	0.914	2 (66.7%)	12 (35.3%)	0.478
II	2 (22.2%)	18 (20.9%)		1 (33.3%)	6 (17.6%)	
III	2 (22.2%)	16 (18.6%)		–	6 (17.6%)	
IV	3 (33.3%)	23 (26.7%)		–	10 (29.4%)	
Vascular invasion	0 (0.0%)	19 (22.1%)	0.115	0 (0.0%)	4 (11.8%)	0.529
Alpha-fetoprotein						
< 20 ng/mL	5 (55.6%)	36 (41.9%)	0.642	2 (66.7%)	16 (63.1%)	0.658
≥ 20 ng/mL	4 (44.4%)	40 (46.5%)		1 (33.3%)	14 (41.2%)	
Not available	–	10 (11.6%)			4 (11.8%)	

### Mutational status and clinical course of disease

A total of 37 (8 fresh frozen (FF) and 29 formalin-fixed, paraffin-embedded (FFPE)) tissue samples were analyzed. Three tissue samples (8.1%) were positive for the *CTNNB1* mutation (1 FF, 2 FFPE). At inclusion, plasma samples from 9 patients out of 95 (9.5%) were positive for the *CTNNB1* p.T41A mutation.

Based on a large proportion of samples being *CTNNB1* mutation negative in both plasma and tumor tissue, the agreement between plasma and tissue mutational status was 91.7% (kappa value 0.53 (0.20–0.85), *P* = 0.0007). Adding results from plasma analyses increased the *CTNNB1* mutation detection rate to 13.5% (5/37) in the subgroup of patients with tumor tissue available.

There was no correlation between *CTNNB1* mutation status and mortality (HR 0.71 (0.28–1.81) *P* = 0.47, adjusted for TNM stage, vascular invasion, sex and age).

**Fig. 1:**
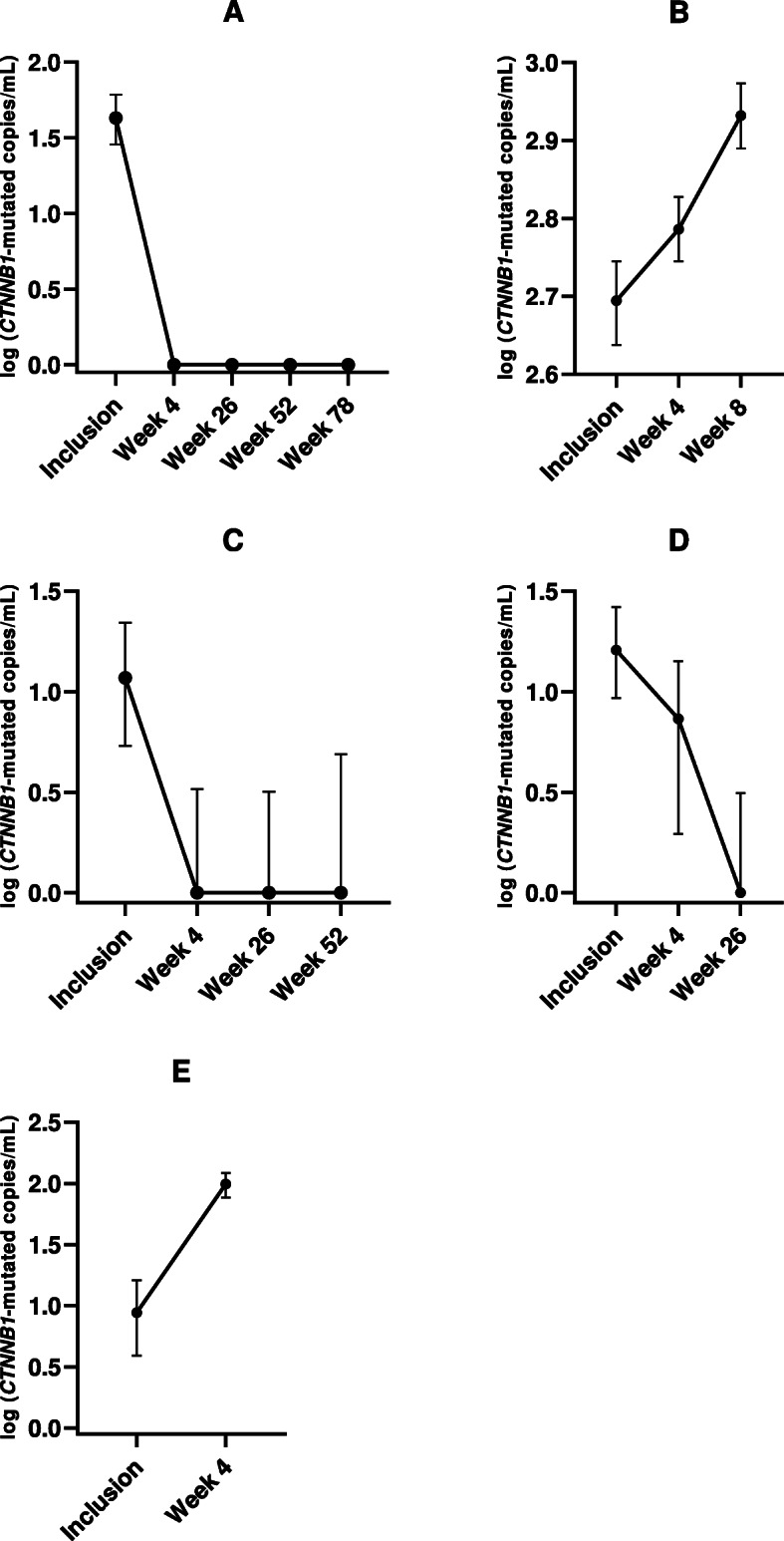
Correlation between amount of *CTNNB1*-mutated alleles with 95% confidence intervals during follow up and clinical course of disease in five HCC patients. **a** Patient underwent resection and concomittant radiofrequency ablation. Relapse diagnosed on CT scans after 1 year. **b** Patient received sorafenib. Stopped treatment after 8 weeks because of progressive disease and declining performance status. **c** Patient underwent radiofrequency ablation with complete response. **d** Patient received Trans Arterial Chemo Embolization with a mixed response. **e** Patient recieved sorafenib. Stopped treatment due to declining performance status. Deceased after 6 months

Figure 1 shows the changes in the concentration of *CTNNB1* mutated alleles during follow up, in relation to treatment and response in the five patients who were positive for *CTNNB1* mutation in plasma at inclusion and with follow-up samples available. The concentration of the mutated alleles correlated to clinical outcome. The patient depicted in Fig. 1a, had two separate intrahepatic tumors on a background of hepatitis C virus, and had a resection of one tumor and radiofrequency ablation of the other tumor. The resected tumor was positive for the *CTNNB1* mutation, while there was no tissue available from the ablated tumor. Mutated alleles were undetectable after treatment. The patient later had recurrent disease at the ablation site, but without detectable *CTNNB1* mutation in plasma in subsequent follow up samples. The patient in Fig. 1b received sorafenib, but treatment was stopped after 8 weeks because of progression and declining performance status. The mutated allele frequency gradually increased during the same period. The patient in Fig. 1c underwent radiofrequency ablation with complete response, mirrored by undetectable levels of mutated alleles in follow up samples. The patient in Fig. 1d received Trans Arterial Chemo Embolization with a mixed clinical response and showed a more delayed decline in mutated allele frequency compared to the complete response observed in patient A and C. And finally, the patient in Fig. 1e received sorafenib, but stopped treatment because of declining performance status and died after 6 months. The clinical course was preceded by a significant increase in mutated alleles within the first 4 weeks of treatment, resembling patient B.

Two patients were positive in plasma, but negative in tumor tissue. In both cases, the FFPE biopsies were from large inhomogeneous tumors (6.2 and 14.5 cm, respectively). Another patient had a positive tissue sample (FFPE) but negative plasma sample at inclusion. Follow up revealed a negative plasma sample at week 4 and week 26 but at week 104 the mutation was detectable, coinciding with progression of disease.

## Discussion

In this study we applied ddPCR to detect the *CTNNB1* p.T41A hotspot mutation in plasma and tumor tissue from HCC patients at inclusion and during 3 years of follow-up. We showed that it is feasible to detect the *CTNNB1* p.T41A mutation in both plasma and tumor samples using ddPCR. In essence, the combined analysis of ctDNA and tumor tissue increased the detection rate of a *CTNNB1* mutation in HCC patients. Moreover, serial testing of ctDNA in individual HCC patients revealed the appearance of *CTNNB1* mutations, possibly reflecting a clonal evolution of HCC or arising multicentric tumors with separate genetic features.

Recent studies suggest that mutations affecting the beta-catenin pathway including the *CTNNB1* p.T41A mutation may prove predictive of beneficial effects of immunotherapy [12, 18, 19]. The *CTNNB1* p.T41A mutation activates the Wnt pathway and leads to T-cell exhaustion and innate resistance to immune checkpoint inhibitors [19, 20]. Harding et al. found a survival difference dependent on Wnt/β pathway mutational status of 6.1 months in patients treated with immune checkpoint inhibitors [12]. This underlines the importance of awareness of the mutational status in the individual patient. Furthermore, adenomas with mutations in the Wnt/β-catenin pathway have higher risk of malignant transformation [21], therefore this noninvasive strategy may also guide decisions regarding treatment or surveillance.

We observed no difference in mortality depending on *CTNNB1* mutational status, however, this study was not designed to evaluate the efficacy of systemic treatment. In five patients with detectable *CTNNB1* mutation in plasma at inclusion, the dynamic changes in the concentration of mutated alleles correlated to treatment response. As a proof-of-concept, ctDNA became undetectable in patients radically treated, and conversely, we observed an increasing number of mutated alleles in patients with progressive disease. This should, however, be investigated in larger studies.

At inclusion, we detected the *CTNNB1* p.T41A mutation in plasma in 9.5% of patients and the mutation rate was 13.5% when combining results from plasma and tumor tissue analysis. Thus, the mutation detection rates are in line with the expected mutation rate demonstrated in previous studies of *CTNNB1* p.T41A mutations in HCC patients [9, 22]. *CTNNB1* mutations are more frequent in alcohol-related HCC [23] and associated with old age and negatively associated with hepatitis B and elevated alpha-fetoprotein [9], thereby mirroring the etiologic profile of the northern European HCC population. However, the number of patients included in this study does not allow validation of these associations.

The majority of previous studies on molecular profiles of HCC were performed on early-stage tumors and without real-time evaluation of impact on treatment effect. ddPCR is a non-invasive method for *CTNNB1* p.T41A mutation that is implementable in most larger HCC treatment facilities. ddPCR may supplement next generation sequencing of tumor tissue samples, as it requires a relatively low input of ctDNA, results are readily interpretable, and ctDNA reflects the genetic background of the entire tumor burden [24]. Importantly, in this study we detected the *CTNNB1* mutation in plasma in two patients in whom tissue biopsy was mutation negative. ddPCR is useful for serial testing of tumor mutational status, as demonstrated by one patient with a positive tissue biopsy who became ctDNA positive during follow-up.

The concordance between plasma and tissue mutational status was high, mainly due to a high number of *CTNNB1* mutation double negative patients. Discrepancy between mutational status in tissue and plasma may be due to tumor heterogeneity, tumor clonal evolution or multicentric tumors, and additionally, different DNA isolation techniques from blood and tissue might have influenced the concordance rate. In one case of discrepancy, with *CTNNB1* mutation in ctDNA while not present in the index biopsy, we observed progression in satellite tumors during follow-up. Together with the results from the patient in Fig. 1a, this scenario could be explained by the presence of multicentric tumors with separate genetic composition.

The strength of the present study is the number of well characterized patients with HCC from a single tertiary referral center and the follow-up of patients during different treatment scenarios. A weakness is the number of tumor tissue available for analysis, mainly due to the international guidelines where the HCC diagnosis in cirrhosis patients can be based exclusively on imaging. This study focused on only the most frequent mutation known in the *CTNNB1* gene. For a more comprehensive characterization, the multiplex ddPCR method may help increase the number of different mutations investigated.

## Conclusions

In conclusion, analysis of ctDNA revealed tumor mutations that were not apparent in single tumor biopsies, and the combined analysis of ctDNA and tumor tissue increased the detection rate of *CTNNB1* mutation in HCC patients. Serial analysis of ctDNA may facilitate a non-invasive personalized therapy strategy by close monitoring of tumor mutation profiles. We suggest larger studies to further explore whether changes in ctDNA can predict and help monitor treatment response in HCC patients.

## Supplementary Information

**Additional file 1.** Droplet digital PCR analysis.

## Data Availability

The data for the study is available upon request to the corresponding author.

## Abbreviations

cfDNA: Cell-free DNA
ctDNA: Circulating tumor DNA
ddPCR: Droplet digital polymerase chain reaction
FFPE: Formalin-fixed paraffin-embedded
HCC: Hepatocellular carcinoma
LoD: Limit of detection
WT: Wild-type
