# Supplementary material for: Combining tissue and circulating tumor DNA increases the detection rate of a CTNNB1 mutation in hepatocellular carcinoma
Source: BMC Cancer. 2021 Apr 8;21:376. doi: 10.1186/s12885-021-08103-0 (PMC8028749; doi:10.1186/s12885-021-08103-0)
Supplement: Supplementary file 1 — Additional file 1. Droplet digital PCR analysis. [file 12885_2021_8103_MOESM1_ESM.docx]

**Droplet digital PCR analysis**

ddPCR analyses were performed using the QX200 AutoDG Droplet Digital PCR system (Bio-Rad, Hercules, CA, USA). The CTNNB1 p.T41A commercial assay from Bio-Rad was used and validated by testing plasma with the cell line A-427. Each ddPCR reaction was run in a total volume of 22 µl including 2× ddPCR Supermix for Probes (No dUTP) (Bio-Rad), the CTNNB1 assay (p.T41A and WT) and 9 µl purified cfDNA following the manufacturer’s protocol. PCR amplification was performed using the following cycling conditions: 95°C for 10 minutes, 40 cycles of 96°C for 30 seconds and 62°C for 1 minute, 98°C for 10 minutes, and 4°C for infinite hold. Samples were run in a minimum of triplicates. Each run included a nontemplate control, cfDNA from a healthy donor and a mutation-positive control. QuantaSoft analysis software version v.1.7.4.0917 (Bio-Rad) was used in all analyses.
The analysis of FFPE tissue was performed by adding 1 U uracil-DNA glycosylase UDG (NEB, Ipswich, MA, USA) to each reaction, and the generated droplets were incubated at 37°C for 20 minutes before PCR cycling.
The limit of detection (LoD) was determined as previously described by analyzing cfDNA from healthy donors^1^. Since the sensitivity, and therefore the LoD, varies with the input of DNA analyzed, we evaluated the LoD for the amount of input DNA in each individual sample. Based on the average wild-type (WT) counts, the LoD of *CTNNB1* p.T41A in plasma is 0.05% (based on 10,650 WT molecules).

1. Milbury CA, Zhong Q, Lin J, et al. Determining lower limits of detection of digital PCR assays for cancer-related gene mutations. *Biomolecular detection and quantification.* 2014;1(1):8-22. <http://dx.doi.org/10.1016/j.bdq.2014.08.001>.
